# Candidate Gene Analysis of Mortality in Dialysis Patients

**DOI:** 10.1371/journal.pone.0143079

**Published:** 2015-11-20

**Authors:** Tonia C. Rothuizen, Gurbey Ocak, Jeffrey J. W. Verschuren, Friedo W. Dekker, Ton J. Rabelink, J. Wouter Jukema, Joris I. Rotmans

**Affiliations:** 1 Department of Nephrology, Leiden University Medical Center, Leiden, The Netherlands; 2 Einthoven Laboratory for Experimental Vascular Medicine, Leiden University Medical Center, Leiden, The Netherlands; 3 Department of Clinical Epidemiology, Leiden University Medical Center, Leiden, The Netherlands; 4 Department of Cardiology, Leiden University Medical Center, Leiden, The Netherlands; 5 Durrer Center for Cardiogenetic Research, Amsterdam, The Netherlands; 6 The Interuniversity Cardiology Institute (ICIN), Utrecht, The Netherlands; University of Florida, UNITED STATES

## Abstract

**Background:**

Dialysis patients have high cardiovascular mortality risk. This study aimed to investigate the association between SNPs of genes involved in vascular processes and mortality in dialysis patients.

**Methods:**

Forty two SNPs in 25 genes involved in endothelial function, vascular remodeling, cell proliferation, inflammation, coagulation and calcium/phosphate metabolism were genotyped in 1330 incident dialysis patients. The effect of SNPs on 5-years cardiovascular and non-cardiovascular mortality was investigated.

**Results:**

The mortality rate was 114/1000 person-years and 49.4% of total mortality was cardiovascular. After correction for multiple testing, *VEGF rs699947* was associated with all-cause mortality (HR1.48, 95% CI 1.14–1.92). The other SNPs were not associated with mortality.

**Conclusions:**

This study provides further evidence that a SNP in the *VEGF* gene may contribute to the comorbid conditions of dialysis patients. Future studies should unravel the underlying mechanisms responsible for the increase in mortality in these patients.

## Introduction

Patients with end stage renal disease (ESRD) have a very high mortality risk as compared with the general population. Cardiovascular disease is a major cause of death in these patients, accounting for 40–50% of total mortality[1,2]. Recently, a large study showed that patients on chronic dialysis had an 8.8-times increased cardiovascular mortality risk as compared with the general population[3]. In addition to cardiovascular disease, declined kidney function and chronic kidney disease (CKD) are associated with increased hospitalization[4], infection[3,5,6], malignancies[7–9] and frailty[10] resulting in an 8.1-fold increased risk of non-cardiovascular mortality[3]. The latter illustrates the very high risk of both cardiovascular and non-cardiovascular death in these patients[3,3,6,11].

These increased mortality rates in ESRD patients are only in part explained by traditional risk factors, suggesting a role for CKD-related factors. CKD specific risk factors include chronic inflammatory state[12] and altered levels of circulating growth factors[13], the presence of uremic toxins[14], disturbed calcium/phosphate metabolism and coagulation[15] as well as endothelial dysfunction[16]. Alterations in the genetic profile of these processes in ESRD patients may further increase this dysbalance and enhance morbidity and mortality.

Interestingly, single nucleotide polymorphisms (SNPs) that influence the above mentioned processes have already been related to coronary restenosis[17–24] and vascular aneurysm formation[25,26] in the general population and to hemodialysis arteriovenous access failure[27–29] by changing vascular function through processes related to endothelial function and vascular remodeling, growth factors, inflammation, coagulation, and calcium/phosphate metabolism[20,24–31]. Thus far, the association between these SNPs and cardiovascular mortality has not been investigated in dialysis patients. Despite their strong cardiovascular link, these SNPs may not be exclusively related to cardiovascular morbidity and mortality. Indeed, the genes affected by these SNPs mediate a plethora of processes, and thus may also affect non-cardiovascular morbidity and mortality. Therefore, we hypothesized that SNPs involved in processes related to endothelial function, vascular remodeling, cell proliferation, inflammation, coagulation, and calcium/phosphate metabolism could influence cardiovascular and non-cardiovascular mortality in patients on dialysis. This study was performed in a large population of incident dialysis patients.

## Subjects and Methods

### Patients

The Netherlands Cooperative Study on the Adequacy of Dialysis (NECOSAD) is a prospective multicenter cohort study in which incident adult ESRD patients from 38 dialysis centers in the Netherlands were included[32]. The study was performed in accordance with the Declaration of Helsinki. The Medical Review Ethics Committee of the Leiden University Medical Center approved the study. All patients gave written informed consent. Adult patients that did not receive any prior renal replacement therapy were eligible. Patients were followed from January 1997 until death or censoring, i.e. transfer to a nonparticipating dialysis center, withdrawal from the study, transplantation or end of the follow-up period (June 2009). Data on dialysis modality, age, sex, and primary kidney disease were collected at the start of dialysis treatment. Primary kidney disease was classified according to the codes of the European Renal Association-European Dialysis and Transplant Association (ERA-EDTA)[33]. Patients were grouped into four classes of primary kidney disease: glomerulonephritis, diabetes mellitus, renal vascular disease and other kidney diseases. Other kidney diseases consisted of patients with interstitial nephritis, polycystic kidney diseases and kidney failure due to multisystem diseases. All-cause mortality was further subdivided in cardiovascular and non-cardiovascular mortality. Cardiovascular death was defined as death due to heart failure, myocardial infarction, ischemic or hemorrhagic stroke, sudden death without obvious non-cardiovascular cause, and death due to other cardiovascular causes. Non-cardiovascular death included all other causes of death.

### SNP selection and genotyping

SNPs of interest were selected that could influence mortality risk by changing vascular processes in dialysis patients. Therefore, SNPs previously associated with vascular disease such as coronary restenosis, arteriovenous (AV) access failure and vascular aneurysm formation were selected after a systematic search of literature. Searching MEDLINE using keywords including ‘hemodialysis’, ‘single nucleotide polymorphism’, ‘arteriovenous access failure’, ‘coronary restenosis’, ‘percutaneous coronary intervention’ and ‘aortic aneurysm’ 42 SNPs in 25 candidate genes were identified[17–29,31,34–55]. Only SNPs with a minor allele frequency higher than 1% were included. The complete list of these candidate genes with associated outcomes is described elsewhere[32]. Two multiplex assays were designed using Assay designer software. When a SNP did not fit the multiplex, a proxy of that SNP was selected with the highest R^2^ value. The final set included 42 SNPs in 25 genes related to growth factors[18,24,26,34–36,38–42] (S1 Table), inflammation[20,21,23,28,43–46] (S2 Table), endothelial function and vascular remodeling[17,27,55] (S3 Table), calcium/phosphate metabolism[22,24,29,47,48] (S4 Table) and coagulation[24,31,50,51,53,54] (S5 Table). All SNPs were genotyped by MALDI-TOF mass spectrometry, using the MassARRAY^tm^ methodology (Sequenom Inc., San Diego, CA, USA), following manufacturer's instructions. As quality control, 5% of the samples were genotyped in duplicate. No inconsistencies were observed. All the negative controls (2%) were negative.

### Statistical analysis

Continuous variables are presented as median and interquartile range (IQR). Categorical variables are presented as number with percentages. Minor allele frequencies were calculated and a chi-squared test with 1 degree freedom was used to determine if observed and expected genotypes were in Hardy Weinberg equilibrium (HWE), using a p-value cut-off of <0.01, to reduce the likelihood of false positivity[32]. The hazard ratios (HRs) with 95% confidence intervals (95% CIs) were calculated using Cox regression analysis for heterozygote genotypes and homozygous mutant genotypes as compared with wild-type genotypes for five-year mortality for the 42 SNPs. All analyses were performed using SPSS statistical software version 20.0 (SPSS, Chicago, Ill, USA). To adjust for multiple testing, the false discovery rate (FDR) was calculated using the method of Benjamini and Hochberg[56]. Although no universal FDR significance threshold has been defined, a cut-point of 0.20 has been suggested for candidate gene association studies, meaning that one should expect at most 20% of declared discoveries to be false[57]. Therefore a cutoff-point of 0.20 was chosen which resulted in a corrected level of significance of p = 0.0048 instead of p = 0.05.

## Results

A total of 1330 dialysis patients were genotyped for the 42 SNPs. Baseline characteristics of the patients are shown in Table 1. The median age was 62.2 years, 39.0% was female, and 14.3% had diabetes mellitus as their primary kidney disease. In addition, approximately 8% of the patients had diabetes as co-morbidity. Of the 1330 patients, 474 (35.6%) died within five years of dialysis treatment. The overall mortality rate was 114 per 1000 person-years. Cardiovascular mortality accounted for 234 of these deaths (49.4%), whereas 50.6% of total mortality was due to non-cardiovascular causes (Table 2).

**Table 1:** Baseline characteristics.

		N = 1330
**Age (years, IQR)**	62.2	50.0–71.8
**Sex, female (n, %)**	515	39.0%
**Race, white (n, %)**	1130	91.4%
**Dialysis modality, hemodialysis (n, %)**	812	64.2%
**Primary kidney disease (n, %)**		
***Diabetes mellitus***	189	14.3%
***Glomerulonephritis***	149	11.3%
***Renal vascular disease***	206	15.6%
***Others***	776	58.8%

**Table 2:** Cardiovascular and non-cardiovascular mortality.

		N	%
**Cardiovascular**	Myocardial infarction	41	8.7
	Heart failure	29	6.1
	Cerebrovascular accident	28	3.8
	Sudden death	41	8.7
	Other	105	22.1
	Total cardiovascular	234	49.4
**Non-cardiovascular**	Infection	56	11.8
	Withdrawal	33	7.0
	Suicide/refusal treatment	59	12.4
	Malignancy	31	6.5
	Other	61	12.9
	Total non-cardiovascular	240	50.6

Minor allele frequencies and HWE p-values are summarized in S6 Table. Three SNPs were not in equilibrium: *vitamin D receptor (VDR) rs4516035*, *interleukin-10 rs1800896* and *TGF-β receptor1 rs1626340*. Notably, none of these SNPs were significantly associated with mortality after correction for multiple testing.

### SNPs and mortality

In total, 42 SNPs in 25 genes involved in vascular processes (endothelial function and vascular remodeling, growth factors, inflammation, coagulation, and calcium/phosphate metabolism) were genotyped. Without correction for multiple testing, three SNPs were associated with cardiovascular mortality. *Vascular endothelial growth factor* (*VEGF) rs2010963* (HR0.62; 95% CI 0.38–1.00) and *tumor necrosis factor rs1799964* (HR0.27; 95% CI 0.10–0.73) were associated with a decreased cardiovascular mortality, while *VEGF rs699947* (HR1.52; 95% CI 1.07–2.17) resulted in an increased risk. In addition, without correction for multiple testing, *matrix metalloproteinase-*1 *rs11292517* (HR0.67; 95% CI0.46–0.99) and *VDR rs2238135* (HR0.33; 95% CI0.13–0.80) were associated with decreased risk of non-cardiovascular mortality, while *rs9804922* (HR3.14; 95% CI1.17–8.46) in an intergenic region on *12q23*.*2*, *CD180 rs5744478* (HR3.25; 95% CI1.34–7.91) and *interleukin-6 rs1800795* (HR1.52; 95% CI1.02–2.25) were associated with an increased non-cardiovascular mortality risk.

**Fig 1:**
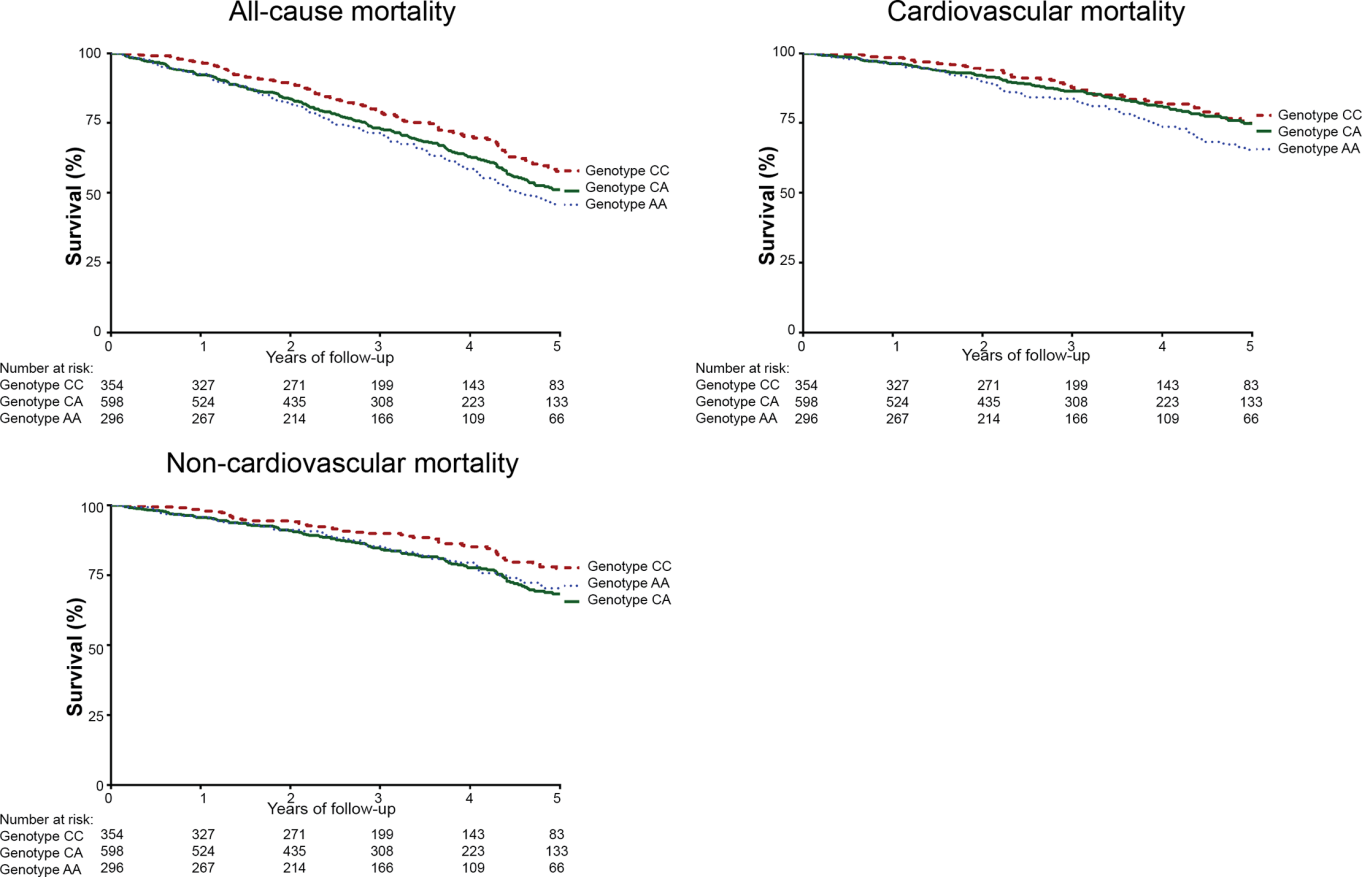
Kaplan Meier survival curve for all-cause, cardiovascular and non-cardiovascular mortality for *VEGF rs699947*.

However, after correction for multiple testing, *VEGF rs699947* only remained significantly associated with all-cause mortality (HR1.48, 95% CI 1.14–1.92, p = 0.00*3)*. Kaplan Meier curves for *VEGF rs699947* are depicted in Fig 1. The results of all other SNPs are summarized in S1–S5 Tables.

## Discussion

Although it is widely recognized that patients on dialysis have substantially higher cardiovascular and non-cardiovascular mortality rates compared with the general population, little is known about the genetic predisposition to mortality of these vulnerable patients. In the present study, we investigated the association between mortality of chronic dialysis patients and 42 SNPs in 25 genes that have previously been linked to cardiovascular disease. We showed that, after correction for multiple testing, *VEGF rs699947* was associated with an increased all-cause mortality risk. This emphasizes that this SNP is not exclusively associated with cardiovascular mortality, but also influences non-cardiovascular mortality. In concordance with previous studies[3,6,58], we observed that the burden of cardiovascular mortality was comparable with non-cardiovascular mortality in our cohort.

The *VEGFA* gene is located on chromosome 6 and is composed of a 14kb coding region with 8 exons and 7 introns[59]. *VEGF rs699947* is situated in the promoter region and can thereby influence VEGF expression levels. Although we did not measure VEGF levels in our study, the effect of the *rs699947* SNP in the *VEGF* gene on VEGF protein levels is reported in other studies. Indeed, carriers of the mutant *A*-allele of *rs699947* on peritoneal dialysis have reduced serum VEGF levels[41]. Additional support for a detrimental effect of *rs699947* SNP of the *VEGF* gene comes from *in vitro* studies which revealed that peripheral blood mononuclear cells isolated from healthy controls produced significantly more VEGF when compared to mononuclear cells from subjects with the *AA* genotype[60]. In our study the mutant *AA* genotype was associated with all-cause mortality, suggesting this can be attributed to both cardiovascular and non-cardiovascular causes. VEGF is involved in angiogenesis, arteriogenesis, vascular permeability, and endothelial cell migration and proliferation[61]. As such, VEGF plays a pivotal role in cardiovascular homeostasis and dysregulation can result in cardiovascular disease. VEGF mediated angiogenesis is important in hypoxic situations such as myocardial infarction since adequate vascular collaterals can preserve the myocardium during ischemia[62] and decrease cardiovascular events[63]. Indeed, carriers of the *AA* genotype of *rs699947*, associated with low levels of VEGF, were shown to have an increased risk of developing coronary artery atherosclerosis[64,65]. In addition to cardiovascular disease, the *rs699947* SNP has also been associated to non-cardiovascular disease and mortality. *AA* carriers were shown to have an increased risk for thyroid cancer[66] and prostate cancer[67]. Moreover, patients with non-small cell lung cancer and *AA* genotype had poorer survival[68]. In addition to malignancies, *VEGF rs699947* is also associated to other non-cardiovascular pathophysiology. Despite low systemic levels of VEGF, patients on peritoneal dialysis with the *AA* genotype expressed high mRNA VEGF levels in their peritoneal dialysis effluent as compared to the *CC* genotype, which was associated with progressive increase in peritoneal transport and even increased mortality[41].

Additional support for the detrimental effects of low VEGF levels comes from non-genetic studies, which revealed that reduced VEGF levels are associated with renal podocyte loss in diabetic nephropathy and progression of renal disease[69]. In addition, selective inhibition of VEGF with bevacuzimab, a monoclonal antibody against VEGF used in oncology, can induce hypertension and proteinuria[70]. Furthermore, females with were shown to have elevated levels of soluble VEGF receptor-1, an endogenous VEGF antagonist [71].

Next to reduced levels of VEGF, very high VEGF levels have been reported to be detrimental as well. Indeed, previous studies demonstrated that highly elevated VEGF levels increase all-cause mortality risk in ESRD patients[13,72]. Besides its pro-angiogenic actions, VEGF can exert pro-inflammatory effects[72] by enhancing vascular permeability and inducing leukocyte adhesion molecules[73]. These data suggest that a dysbalance in VEGF levels, either decreased or largely increased, may potentially be pathogenic.

Our study has several potential limitations. The collective term cardiovascular disease comprises a plethora of disorders elicited by even more underlying processes. We investigated SNPs involved in endothelial function, vascular remodeling, cell proliferation, inflammation, coagulation and calcium/phosphate metabolism, as these processes play an important role in cardiovascular disease. Importantly, these processes are affected in CKD, and alterations in their genetic profile may further increase this dysbalance and enhance morbidity and mortality. Nonetheless, more mechanisms are involved in the broad scope of cardiovascular disease and the selection of SNPs in this article is not exhaustive. For example, polymorphisms in iron metabolism and vascular calcification are missing, while these could be relevant in a dialysis population. Future studies could elaborate on the current selection. In addition, we primarily selected the SNPs on their cardiovascular interactions. Because the genes affected by these SNPs also exert important non-cardiovascular effects and dialysis patients also suffer from a large burden of non-cardiovascular mortality[3], we did not want to neglect this and also investigated their influence on non-cardiovascular mortality. However, other SNPs that may contribute to non-cardiovascular mortality were not appraised in this study. Considering the increasing attention to non-cardiovascular mortality in ESRD patients[74], future studies should investigate the effects of other SNPs primarily influencing non-cardiovascular disease.

Secondly, despite the large size of our cohort, in some SNPs there was a very small sample size in especially the variant genotypes. These groups are likely insufficiently powered to detect an association and this may lead to underestimation of the actual effect of the SNPs.

Furthermore, we did not measure plasma VEGF levels in this study. However, previous studies convincingly demonstrated decreased VEGF levels in the *rs699947 AA* genotype in both healthy individuals[60] and dialysis patients[41], thereby providing support for our assumption that the increased mortality as observed in dialysis patients carrying the *VEGF rs699947* SNP, might be explained by decreased serum VEGF levels.

In conclusion, this study provides evidence that *VEGF rs699947 AA* genotype is associated with all-cause mortality in a large cohort of dialysis patients, whereas there was no significant association with the other 41 SNPs. These results may help clarify the involved pathways in the increased mortality of these patients. Further studies should investigate the underlying mechanisms in order to develop new therapies aimed to reduce the dramatic mortality rates in dialysis patients. In addition, stratification of patients with genetic risk factors combined with clinical risk factors could be used to predict mortality for specific subgroups of dialysis patients and may facilitate tailored therapies.

## Supporting Information

S1 Table Polymorphisms in growth factor related genes and effect on five-years mortality. GT, genotype; SNP, single nucleotide polymorphism; N, number of subjects; HR, hazard ratio; CI confidence interval; NE, not estimable.
(DOC)

S2 Table Polymorphisms related to inflammatory genes and effect on five-years mortality. GT, genotype; SNP, single nucleotide polymorphism; N, number of subjects; HR, hazard ratio; CI confidence interval; NE, not estimable.
(DOC)

S3 Table Polymorphisms related to endothelial function and vascular remodeling and effect on five-years mortality. GT, genotype; SNP, single nucleotide polymorphism; N, number of subjects; HR, hazard ratio; CI confidence interval; NE, not estimable.
(DOC)

S4 Table Polymorphisms related to calcium/phosphate metabolism and effect on five-years mortality. * rs397703 is a proxy for rs1207568 (R^2^ = 0.70). GT, genotype; SNP, single nucleotide polymorphism; N, number of subjects; HR, hazard ratio; CI confidence interval; NE, not estimable.
(DOC)

S5 Table Polymorphisms related to coagulation and effect on five-years mortality. * rs1800787 was a proxy for rs1800790 (R^2^ = 1.0). ^†^ rs1718711 was a proxy for rs5918 (R^2^ = 0.93). GT, genotype; SNP, single nucleotide polymorphism; N, number of subjects; HR, hazard ratio; CI, confidence interval.
(DOC)

S6 Table MAFs and HWE p-values. MAF, minor allele frequency, HWE p-value, Hardy Weinberg equilibrium χ^2^ test p-values. P-value <0.05 suggests a disequilibrium.
(DOC)
