# Supplementary material for: Candidate Gene Analysis of Mortality in Dialysis Patients
Source: PLoS One. 2015 Nov 20;10(11):e0143079. doi: 10.1371/journal.pone.0143079 (PMC4654483; doi:10.1371/journal.pone.0143079)
Supplement: S1 Table — GT, genotype; SNP, single nucleotide polymorphism; N, number of subjects; HR, hazard ratio; CI confidence interval; NE, not estimable. (DOC) [file pone.0143079.s001.doc]

**S1 Table. Polymorphisms in growth factor related genes and effect on five-years mortality**

| **Gene** | **Name** | **SNP** | **GT** | **N** | **All-Cause** | | | **Non-Cardiovascular** | | | **Cardiovascular** | | |
| --- | --- | --- | --- | --- | --- | --- | --- | --- | --- | --- | --- | --- | --- |
|  |  |  |  |  | **HR (95% CI)** | | **P** | **HR (95% CI)** | | **P** | **HR (95% CI)** | | **P** |
| CDKN1B | Cyclin-dependent kinase inhibitor 1B | rs36228499 | CC | 400 | 1 | Ref |  | 1 | Ref |  | 1 | Ref |  |
| (p27kip1) |  |  | CA | 623 | 1.06 | 0.86-1.30 | 0.61 | 1.20 | 0.89-1.62 | 0.24 | 0.93 | 0.69-1.25 | 0.63 |
|  |  |  | AA | 242 | 1.02 | 0.78-1.34 | 0.88 | 1.09 | 0.74-1.60 | 0.68 | 0.97 | 0.66-1.41 | 0.86 |
| CTGF | Connective Tissue Growth Factor | rs6918698 | CC | 377 | 1 | Ref |  | 1 | Ref |  | 1 | Ref |  |
|  |  |  | CG | 598 | 0.92 | 0.74-1.15 | 0.47 | 1.01 | 0.74-1.36 | 0.99 | 0.84 | 0.61-1.16 | 0.29 |
|  |  |  | GG | 284 | 1.22 | 0.95-1.56 | 0.11 | 1.09 | 0.76-1.57 | 0.63 | 1.35 | 0.96-1.90 | 0.09 |
| FGFR4 | Fibroblast Growth Factor Receptor 4 | rs351855 | GG | 573 | 1 | Ref |  | 1 | Ref |  | 1 | Ref |  |
|  |  |  | GA | 495 | 1.09 | 0.89-1.33 | 0.41 | 1.12 | 0.84-1.49 | 0.46 | 1.06 | 0.80-1.41 | 0.76 |
|  |  |  | AA | 142 | 1.07 | 0.79-1.45 | 0.68 | 1.28 | 0.85-1.94 | 0.23 | 0.87 | 0.55-1.38 | 0.55 |
| KLF5 | Kruppel-like Factor 5 | rs3812852 | AA | 1088 | 1 | Ref |  | 1 | Ref |  | 1 | Ref |  |
|  |  |  | AG | 179 | 1.00 | 0.77-1.30 | 0.99 | 1.02 | 0.71-1.48 | 0.91 | 1.00 | 0.67-1.43 | 0.90 |
|  |  |  | GG | 5 | NE |  |  | NE |  |  | NE |  |  |
| PDGFD | Platelet Derived Growth Factor D | rs974819 | CC | 601 | 1 | Ref |  | 1 | Ref |  | 1 | Ref |  |
|  |  |  | CT | 534 | 0.77 | 0.64-1.94 | 0.009 | 0.84 | 0.64-1.10 | 0.20 | 0.71 | 0.53-0.94 | 0.02 |
|  |  |  | TT | 132 | 0.74 | 0.53-1.03 | 0.070 | 0.65 | 0.40-1.07 | 0.09 | 0.82 | 0.52-1.28 | 0.37 |
| PDGFD | Platelet Derived Growth Factor D | rs496339 | AA | 1019 | 1 | Ref |  | 1 | Ref |  | 1 | Ref |  |
|  |  |  | AG | 228 | 1.25 | 0.99-1.57 | 0.06 | 1.24 | 0.90-1.71 | 0.20 | 1.26 | 0.91-1.74 | 0.17 |
|  |  |  | GG | 13 | 0.86 | 0.32-2.30 | 0.76 | 1.27 | 0.41-3.97 | 0.68 | 0.44 | 0.06-3.12 | 0.41 |
| TGFBR1 | Transforming Growth Factor β Receptor 1 | rs1626340 | GG | 781 | 1 | Ref |  | 1 | Ref |  | 1 | Ref |  |
|  |  |  | GA | 368 | 0.97 | 0.79-1.19 | 0.76 | 0.95 | 0.71-1.27 | 0.73 | 0.99 | 0.74-1.32 | 0.93 |
|  |  |  | AA | 67 | 0.64 | 0.40-1.02 | 0.006 | 0.81 | 0.45-1.46 | 0.48 | 0.47 | 0.22-1.01 | 0.054 |
| TGFBR2 | Transforming Growth Factor β Receptor 2 | rs1036095 | GG | 716 | 1 | Ref |  | 1 | Ref |  | 1 | Ref |  |
|  |  |  | GC | 468 | 0.92 | 0.75-1.11 | 0.38 | 1.02 | 0.78-1.34 | 0.86 | 0.82 | 0.62-1.08 | 0.16 |
|  |  |  | CC | 77 | 0.85 | 0.56-1.29 | 0.44 | 1.11 | 0.65-1.89 | 0.71 | 0.61 | 0.31-1.20 | 0.15 |
| TGFBR2 | Transforming Growth Factor β Receptor 2 | rs4522809 | AA | 381 | 1 | Ref |  | 1 | Ref |  | 1 | Ref |  |
|  |  |  | AG | 633 | 0.95 | 0.77-1.18 | 0.65 | 1.11 | 0.82-1.50 | 0.49 | 0.82 | 0.61-1.10 | 0.18 |
|  |  |  | GG | 245 | 1.00 | 0.77-1.31 | 0.98 | 1.07 | 0.73-1.57 | 0.72 | 0.94 | 0.65-1.36 | 0.75 |
| VEGF | Vascular Endothelial Growth Factor | rs2010963 | GG | 566 | 1 | Ref |  | 1 | Ref |  | 1 | Ref |  |
|  |  |  | GC | 538 | 0.87 | 0.72-1.06 | 0.17 | 0.83 | 0.63-1.09 | 0.17 | 0.92 | 0.70-1.21 | 0.56 |
|  |  |  | CC | 157 | 0.75 | 0.55-1.02 | 0.06 | 0.87 | 0.58-1.31 | 0.50 | 0.62 | 0.38-1.00 | 0.05 |
| VEGF | Vascular Endothelial Growth Factor | rs3025039 | CC | 958 | 1 | Ref |  | 1 | Ref |  | 1 | Ref |  |
|  |  |  | CT | 291 | 0.91 | 0.72-1.13 | 0.38 | 0.82 | 0.59-1.13 | 0.22 | 1.00 | 0.74-1.36 | 1.00 |
|  |  |  | TT | 25 | 1.32 | 0.70-2.47 | 0.39 | 1.02 | 0.38-2.73 | 0.98 | 1.64 | 0.73-3.70 | 0.23 |
| VEGF | Vascular Endothelial Growth Factor | rs699947 | CC | 354 | 1 | Ref |  | 1 | Ref |  | 1 | Ref |  |
|  |  |  | CA | 598 | 1.29 | 1.02-1.63 | 0.03 | 1.51 | 1.08-2.10 | 0.02 | 1.10 | 0.79-1.53 | 0.50 |
|  |  |  | AA | 296 | 1.48 | 1.14-1.92 | 0.003 | 1.43 | 0.98-2.09 | 0.07 | 1.52 | 1.07-2.17 | 0.02 |

GT, genotype; SNP, single nucleotide polymorphism; N, number of subjects; HR, hazard ratio; CI confidence interval; NE, not estimable.
