# Supplementary material for: Candidate Gene Analysis of Mortality in Dialysis Patients
Source: PLoS One. 2015 Nov 20;10(11):e0143079. doi: 10.1371/journal.pone.0143079 (PMC4654483; doi:10.1371/journal.pone.0143079)
Supplement: S2 Table — GT, genotype; SNP, single nucleotide polymorphism; N, number of subjects; HR, hazard ratio; CI confidence interval; NE, not estimable. (DOC) [file pone.0143079.s002.doc]

**S2 Table. Polymorphisms related to inflammatory genes** and effect on **five-years mortality**

| **Gene** | **Name** | **SNP** | **GT** | **N** | **All-Cause** | | **Non-Cardiovascular** | | | **Cardiovascular** | | | |
| --- | --- | --- | --- | --- | --- | --- | --- | --- | --- | --- | --- | --- | --- |
|  |  |  |  |  | **HR (95% CI)** | | **P** | **HR (95% CI)** | | **P** | **HR (95% CI)** | | **P** |
| CD180 (RP105) | CD180 | rs5744478 | TT | 1084 | 1 | Ref |  | 1 | Ref |  | 1 | Ref |  |
|  |  |  | TC | 179 | 1.30 | 1.02-1.66 | 0.037 | 1.32 | 0.93-1.86 | 0.12 | 1.28 | 0.91-1.82 | 0.16 |
|  |  |  | CC | 11 | 2.27 | 1.07-4.79 | 0.032 | 3.25 | 1.34-7.91 | 0.009 | 1.29 | 0.32-5.19 | 0.72 |
| IL6 | Interleukin 6 | rs1800795 | GG | 504 | 1 | Ref |  | 1 | Ref |  | 1 | Ref |  |
|  | CD180  (RP105) |  | GC | 585 | 1.17 | 0.96-1.43 | 0.13 | 1.41 | 1.06-1.89 | 0.019 | 0.97 | 0.73-1.28 | 0.81 |
|  |  |  | CC | 183 | 1.32 | 1.00-1.74 | 0.051 | 1.52 | 1.02-2.25 | 0.04 | 1.15 | 0.78-1.71 | 0.48 |
| IL10 | Interleukin 10 | rs1800896 | AA | 367 | 1 | Ref |  | 1 | Ref |  | 1 | Ref |  |
|  |  |  | AG | 384 | 1.06 | 0.85-1.31 | 0.61 | 1.19 | 0.87-1.61 | 0.28 | 0.94 | 0.69-1.28 | 0.70 |
|  |  |  | GG | 306 | 0.97 | 0.75-1.25 | 0.79 | 1.01 | 0.70-1.46 | 0.96 | 0.92 | 0.65-1.32 | 0.66 |
| IL10 | Interleukin 10 | rs3024498 | AA | 682 | 1 | Ref |  | 1 | Ref |  | 1 | Ref |  |
|  |  |  | AG | 478 | 0.98 | 0.81-1.19 | 0.86 | 1.11 | 0.85-1.45 | 0.44 | 0.86 | 0.65-1.14 | 0.30 |
|  |  |  | GG | 103 | 0.88 | 0.62-1.27 | 0.50 | 0.69 | 0.39-1.22 | 0.20 | 1.07 | 0.68-1.70 | 0.76 |
| TNF | Tumor Necrosis Factor | rs1799964 | TT | 740 | 1 | Ref |  | 1 | Ref |  | 1 | Ref |  |
|  |  |  | TC | 449 | 1.09 | 0.90-1.32 | 0.36 | 1.19 | 0.91-1.56 | 0.21 | 1.00 | 0.78-1.32 | 0.96 |
|  |  |  | CC | 73 | 0.60 | 0.37-0.98 | 0.04 | 0.96 | 0.55-1.71 | 0.90 | 0.27 | 0.10-0.73 | 0.01 |
| TNF | Tumor Necrosis Factor | rs1800629 | GG | 855 | 1 | Ref |  | 1 | Ref |  | 1 | Ref |  |
|  |  |  | GA | 362 | 1.34 | 1.10-1.64 | 0.003 | 1.45 | 1.10-1.91 | 0.008 | 1.24 | 0.94-1.65 | 0.13 |
|  |  |  | AA | 46 | 1.24 | 0.76-2.02 | 0.394 | 1.63 | 0.88-3.01 | 0.12 | 0.86 | 0.38-1.94 | 0.71 |
| TNF | Tumor Necrosis Factor | rs361525 | GG | 1144 | 1 | Ref |  | 1 | Ref |  | 1 | Ref |  |
|  |  |  | GA | 113 | 0.90 | 0.65-1.26 | 0.55 | 0.93 | 0.58-1.48 | 0.75 | 0.88 | 0.54-1.42 | 0.60 |
|  |  |  | AA | 6 | 1.70 | 0.64-4.55 | 0.29 | 2.55 | 0.82-7.99 | 0.11 | 0.85 | 0.12-6.06 | 0.87 |
| TLR4 | Toll-Like Receptor 4 | rs4986790 | AA | 1101 | 1 | Ref |  | 1 | Ref |  | 1 | Ref |  |
|  |  |  | AG | 166 | 1.15 | 0.88-1.50 | 0.30 | 0.91 | 0.60-1.37 | 0.64 | 1.42 | 0.99-2.01 | 0.054 |
|  |  |  | GG | 6 | 1.65 | 0.41-6.62 | 0.48 | NE |  |  | 3.50 | 0.87-14.10 | 0.07 |

GT, genotype; SNP, single nucleotide polymorphism; N, number of subjects; HR, hazard ratio; CI confidence interval; NE, not estimable.
