# Supplementary material for: Candidate Gene Analysis of Mortality in Dialysis Patients
Source: PLoS One. 2015 Nov 20;10(11):e0143079. doi: 10.1371/journal.pone.0143079 (PMC4654483; doi:10.1371/journal.pone.0143079)
Supplement: S3 Table — GT, genotype; SNP, single nucleotide polymorphism; N, number of subjects; HR, hazard ratio; CI confidence interval; NE, not estimable. (DOC) [file pone.0143079.s003.doc]

**S3 Table.** **Polymorphisms related to endothelial function and vascular remodeling and effect on five-years mortality**

| **Gene** | **Name** | **SNP** | **GT** | **N** | **All-Cause** | | | **Non-Cardiovascular** | | | **Cardiovascular** | | |
| --- | --- | --- | --- | --- | --- | --- | --- | --- | --- | --- | --- | --- | --- |
|  |  |  |  |  | **HR (95% CI)** | | **P** | **HR (95% CI)** | | **P** | **HR (95% CI)** | | **P** |
| MMP1 | Matrixmetalloproteinase 1 | rs11292517 | 1G/1G | 252 | 1 | Ref |  | 1 | Ref |  | 1 | Ref |  |
|  |  |  | 1G/2G | 608 | 0.96 | 0.77-1.19 | 0.68 | 0.91 | 0.68-1.22 | 0.53 | 1 | 0.74-1.38 | 0.95 |
|  |  |  | 2G/2G | 276 | 0.86 | 0.66-1.12 | 0.26 | 0.67 | 0.46-0.99 | 0.045 | 1.07 | 0.75-1.55 | 0.70 |
| NOS3 | Nitric Oxide Synthase 3 | rs1799983 | GG | 624 | 1 | Ref |  | 1 | Ref |  | 1 | Ref |  |
|  |  |  | GA | 525 | 0.97 | 0.80-1.18 | 0.77 | 0.89 | 0.68-1.17 | 0.40 | 1.06 | 0.81-1.40 | 0.66 |
|  |  |  | AA | 114 | 0.95 | 0.67-1.34 | 0.76 | 0.91 | 0.56-1.47 | 0.69 | 1.00 | 0.61-1.62 | 0.99 |
| ELN | Elastin | rs2071307 | GG | 486 | 1 | Ref |  | 1 | Ref |  | 1 | Ref |  |
|  |  |  | GA | 595 | 1.31 | 1.08-1.60 | 0.008 | 1.45 | 1.10-1.93 | 0.01 | 1.18 | 0.89-1.57 | 0.24 |
|  |  |  | AA | 191 | 0.95 | 0.71-1.29 | 0.76 | 0.96 | 0.62-1.48 | 0.86 | 0.95 | 0.63-1.43 | 0.81 |
| ANXA5 | Annexin A5 | rs4833229 | CC | 415 | 1 | Ref |  | 1 | Ref |  | 1 | Ref |  |
|  |  |  | CT | 608 | 0.89 | 0.72-1.09 | 0.25 | 0.87 | 0.65-1.17 | 0.37 | 0.90 | 0.67-1.20 | 0.46 |
|  |  |  | TT | 238 | 0.95 | 0.74-1.23 | 0.71 | 1.10 | 0.78-1.57 | 0.59 | 0.81 | 0.55-1.18 | 0.27 |
| ANXA5 | Annexin A5 | rs6830321 | GG | 377 | 1 | Ref |  | 1 | Ref |  | 1 | Ref |  |
|  |  |  | GA | 616 | 0.92 | 0.75-1.14 | 0.45 | 0.92 | 0.68-1.24 | 0.58 | 0.93 | 0.69-1.25 | 0.61 |
|  |  |  | AA | 278 | 1.00 | 0.67-1.12 | 0.28 | 1.00 | 0.70-1.43 | 0.99 | 0.74 | 0.51-1.08 | 0.11 |
| LRP1 | Low Density Lipoprotein Receptor Related Protein 1 | rs1466535 | GG | 560 | 1 | Ref |  | 1 | Ref |  | 1 | Ref |  |
|  |  |  | GA | 572 | 0.94 | 0.78-1.15 | 0.56 | 0.94 | 0.71-1.24 | 0.67 | 0.95 | 0.72-1.25 | 0.69 |
|  |  |  | AA | 140 | 1.10 | 0.71-1.31 | 0.79 | 1.10 | 0.73-1.66 | 0.66 | 0.82 | 0.52-1.31 | 0.41 |
| QKI | Quaking | rs3857504 | CC | 856 | 1 | Ref |  | 1 | Ref |  | 1 | Ref |  |
|  |  |  | CT | 355 | 0.98 | 0.80-1.22 | 0.91 | 0.98 | 0.73-1.32 | 0.90 | 1.00 | 0.74-1.34 | 0.98 |
|  |  |  | TT | 30 | 0.90 | 0.51-1.60 | 0.72 | 0.44 | 0.14-1.38 | 0.16 | 1.37 | 0.70-2.69 | 0.36 |
| QKI | Quaking | rs3763197 | TT | 885 | 1 | Ref |  | 1 | Ref |  | 1 | Ref |  |
|  |  |  | TC | 357 | 1.02 | 0.81-1.22 | 0.96 | 1.02 | 0.77-1.36 | 0.88 | 0.97 | 0.72-1.30 | 0.81 |
|  |  |  | CC | 27 | 1.12 | 0.64-1.95 | 0.69 | 0.51 | 0.16-1.59 | 0.25 | 1.76 | 0.93-3.32 | 0.09 |
| QKI | Quaking | rs2759393 | CC | 773 | 1 | Ref |  | 1 | Ref |  | 1 | Ref |  |
|  |  |  | CA | 416 | 1.03 | 0.78-1.16 | 0.62 | 1.03 | 0.78-1.37 | 0.83 | 0.87 | 0.65-1.17 | 0.36 |
|  |  |  | AA | 68 | 1.03 | 0.58-1.35 | 0.57 | 1.03 | 0.58-1.81 | 0.93 | 0.75 | 0.40-1.42 | 0.38 |
| - | 12q23.2 | rs10861032 | TT | 858 | 1 | Ref |  | 1 | Ref |  | 1 | Ref |  |
|  |  |  | TT | 350 | 1.11 | 0.92-1.38 | 0.25 | 1.11 | 0.83-1.48 | 0.49 | 1.15 | 0.86-1.53 | 0.35 |
|  |  |  | CC | 50 | 1.20 | 0.58-1.54 | 0.82 | 1.20 | 0.65-2.22 | 0.55 | 0.68 | 0.30-1.54 | 0.35 |
| - | 12q23.2 | rs9804922 | CC | 1050 | 1 | Ref |  | 1 | Ref |  | 1 | Ref |  |
|  |  |  | CT | 201 | 1.11 | 1.00-1.61 | 0.05 | 1.11 | 0.78-1.58 | 0.55 | 1.43 | 1.03-1.98 | 0.03 |
|  |  |  | TT | 10 | 3.14 | 0.61-4.38 | 0.33 | 3.14 | 1.17-8.46 | 0.02 | NE |  |  |

GT, genotype; SNP, single nucleotide polymorphism; N, number of subjects; HR, hazard ratio; CI confidence interval; NE, not estimable.
