# Supplementary material for: Candidate Gene Analysis of Mortality in Dialysis Patients
Source: PLoS One. 2015 Nov 20;10(11):e0143079. doi: 10.1371/journal.pone.0143079 (PMC4654483; doi:10.1371/journal.pone.0143079)
Supplement: S4 Table — * rs397703 is a proxy for rs1207568 (R2 = 0.70). GT, genotype; SNP, single nucleotide polymorphism; N, number of subjects; HR, hazard ratio; CI confidence interval; NE, not estimable. (DOC) [file pone.0143079.s004.doc]

**S4 Table. Polymorphisms related to calcium/phosphate metabolism and effect on five-years mortality**

| **Gene** | **Name** | **SNP** | **GT** | **N** | **All-Cause** | | | **Non-Cardiovascular** | | | **Cardiovascular** | | |
| --- | --- | --- | --- | --- | --- | --- | --- | --- | --- | --- | --- | --- | --- |
|  |  |  |  |  | **HR (95% CI)** | | **P** | **HR (95% CI)** | | **P** | **HR (95% CI)** | | **P** |
| Klotho | Klotho | rs9527025 | GG | 984 | 1 | Ref |  | 1 | Ref |  | 1 | Ref |  |
|  |  |  | GC | 269 | 0.82 | 0.64-1.04 | 0.10 | 1.17 | 0.86-1.58 | 0.32 | 0.51 | 0.34-0.76 | 0.001 |
|  |  |  | CC | 17 | 0.41 | 0.13-1.27 | 0.12 | 0.29 | 0.04-2.08 | 0.22 | 0.51 | 0.13-2.06 | 0.35 |
| Klotho | Klotho | rs564481 | CC | 464 | 1 | Ref |  | 1 | Ref |  | 1 | Ref |  |
|  |  |  | CT | 571 | 0.95 | 0.78-1.17 | 0.64 | 0.99 | 0.74-1.33 | 0.95 | 0.92 | 0.69-1.22 | 0.55 |
|  |  |  | TT | 201 | 0.98 | 0.74-1.29 | 0.88 | 1.14 | 0.78-1.65 | 0.50 | 0.83 | 0.55-1.24 | 0.37 |
| Klotho | Klotho | rs397703* | TT | 868 | 1 | Ref |  | 1 | Ref |  | 1 | Ref |  |
|  |  |  | TC | 343 | 1.10 | 0.90-1.36 | 0.35 | 1.20 | 0.90-1.59 | 0.22 | 1.01 | 0.75-1.37 | 0.94 |
|  |  |  | CC | 52 | 1.04 | 0.67-1.62 | 0.86 | 0.89 | 0.45-1.74 | 0.73 | 1.20 | 0.67-2.16 | 0.55 |
| Klotho | Klotho | rs577912 | GG | 905 | 1 | Ref |  | 1 | Ref |  | 1 | Ref |  |
|  |  |  | GT | 310 | 1.12 | 0.91-1.38 | 0.30 | 0.99 | 0.73-1.34 | 0.94 | 1.26 | 0.94-1.69 | 0.12 |
|  |  |  | TT | 40 | 1.31 | 0.81-2.10 | 0.27 | 1.11 | 0.54-2.45 | 0.78 | 1.52 | 0.80-2.89 | 0.20 |
| VDR | Vitamin D Receptor | rs11574027 | GG | 1232 | 1 | Ref |  | 1 | Ref |  | 1 | Ref |  |
|  |  |  | GT | 40 | 0.80 | 0.44-1.45 | 0.46 | 0.57 | 0.21-1.54 | 0.27 | 1.03 | 0.48-2.18 | 0.95 |
|  |  |  | TT | 1 | NE |  |  | NE |  |  | NE |  |  |
| VDR | Vitamin D Receptor | rs2238135 | GG | 745 | 1 | Ref |  | 1 | Ref |  | 1 | Ref |  |
|  |  |  | GC | 439 | 1.01 | 0.83-1.23 | 0.94 | 1.00 | 0.76-1.32 | 1.00 | 1.02 | 0.77-1.35 | 0.91 |
|  |  |  | CC | 69 | 0.58 | 0.35-0.94 | 0.03 | 0.33 | 0.13-0.80 | 0.01 | 0.84 | 0.47-1.52 | 0.57 |
| VDR | Vitamin D Receptor | rs4516035 | AA | 425 | 1 | Ref |  | 1 | Ref |  | 1 | Ref |  |
|  |  |  | AG | 567 | 1.01 | 0.82-1.25 | 0.91 | 1.21 | 0.90-1.64 | 0.22 | 0.84 | 0.62-1.14 | 0.27 |
|  |  |  | GG | 260 | 1.09 | 0.84-1.40 | 0.52 | 1.15 | 0.80-1.67 | 0.46 | 1.03 | 0.73-1.47 | 0.86 |
| AHSG | α2-HS Glycoprotein (Fetuin A) | rs4918 | CC | 586 | 1 | Ref |  | 1 | Ref |  | 1 | Ref |  |
|  |  |  | CG | 544 | 1.03 | 0.85-1.25 | 0.78 | 1.09 | 0.83-1.43 | 0.55 | 0.97 | 0.73-1.28 | 0.83 |
|  |  |  | GG | 118 | 1.18 | 0.86-1.62 | 0.30 | 1.15 | 0.73-1.80 | 0.55 | 1.21 | 0.78-1.88 | 0.39 |

* rs397703 is a proxy for rs1207568 (R^2^=0.70). GT, genotype; SNP, single nucleotide polymorphism; N, number of subjects; HR, hazard ratio; CI confidence interval; NE, not estimable.
