# Supplementary material for: Candidate Gene Analysis of Mortality in Dialysis Patients
Source: PLoS One. 2015 Nov 20;10(11):e0143079. doi: 10.1371/journal.pone.0143079 (PMC4654483; doi:10.1371/journal.pone.0143079)
Supplement: S5 Table — * rs1800787 was a proxy for rs1800790 (R2 = 1.0). † rs1718711 was a proxy for rs5918 (R2 = 0.93). GT, genotype; SNP, single nucleotide polymorphism; N, number of subjects; HR, hazard ratio; CI, confidence interval. (DOC) [file pone.0143079.s005.doc]

S5 Table. Polymorphisms related to coagulation and effect on five-years mortality

| **Gene** | **Name** | **SNP** | **GT** | **N** | **All-Cause** | | | **Non-Cardiovascular** | | | | **Cardiovascular** | | |
| --- | --- | --- | --- | --- | --- | --- | --- | --- | --- | --- | --- | --- | --- | --- |
|  |  |  |  |  | **HR (95% CI)** | | **P** | **HR (95% CI)** | | | **P** | **HR (95% CI)** | | **P** |
| FBG | Fibronogen β | rs1044291 | CC | 570 | 1 | Ref |  | 1 | Ref |  | | 1 | Ref |  |
|  |  |  | CT | 542 | 0.94 | 0.77-1.14 | 0.52 | 1.03 | 0.78-1.36 | 0.82 | | 0.85 | 0.64-1.12 | 0.24 |
|  |  |  | TT | 147 | 1.19 | 0.89-1.58 | 0.25 | 1.18 | 0.78-1.79 | 0.43 | | 1.19 | 0.80-1.77 | 0.40 |
| FBG | Fibronogen β | rs1800787* | CC | 789 | 1 | Ref |  | 1 | Ref |  | | 1 | Ref |  |
|  |  |  | CT | 406 | 1.06 | 0.87-1.29 | 0.56 | 1.07 | 0.82-1.42 | 0.61 | | 1.05 | 0.79-1.39 | 0.74 |
|  |  |  | TT | 57 | 1.09 | 0.69-1.74 | 0.71 | 0.80 | 0.38-1.71 | 0.57 | | 1.39 | 0.77-2.51 | 0.28 |
| ITGB3 | Integrin β3  (Platelet Glycoprotein IIIa) | rs17218711^†^ | GG | 924 | 1 | Ref |  | 1 | Ref |  | | 1 | Ref |  |
|  |  |  | GC | 312 | 1.07 | 0.86-1.32 | 0.55 | 1.12 | 0.83-1.50 | 0.46 | | 1.02 | 0.75-1.38 | 0.92 |
|  |  |  | CC | 28 | 0.99 | 0.55-1.81 | 0.98 | 0.90 | 0.37-2.19 | 0.82 | | 1.09 | 0.48-2.46 | 0.84 |

* rs1800787 was a proxy for rs1800790 (R^2^=1.0). ^†^ rs1718711 was a proxy for rs5918 (R^2^=0.93). GT, genotype; SNP, single nucleotide polymorphism; N, number of subjects; HR, hazard ratio; CI, confidence interval.
