# Supplementary material for: Candidate Gene Analysis of Mortality in Dialysis Patients
Source: PLoS One. 2015 Nov 20;10(11):e0143079. doi: 10.1371/journal.pone.0143079 (PMC4654483; doi:10.1371/journal.pone.0143079)
Supplement: S6 Table — MAF, minor allele frequency, HWE p-value, Hardy Weinberg equilibrium χ2 test p-values. P-value <0.05 suggests a disequilibrium. (DOC) [file pone.0143079.s006.doc]

S6 Table. MAFs and HWE p-values

| **SNP** | **Variant allele frequency** | **HWE p-value** |
| --- | --- | --- |
| rs3763197 | 0.16 | 0.194 |
| rs1799983 | 0.30 | 0.812 |
| rs2071307 | 0.38 | 0.687 |
| rs4833229 | 0.43 | 0.563 |
| rs6830321 | 0.46 | 0.377 |
| rs1466535 | 0.33 | 0.737 |
| rs3857504 | 0.17 | 0.339 |
| rs11292517 | 0.51 | 0.017 |
| rs2759393 | 0.22 | 0.223 |
| rs10861032 | 0.18 | 0.061 |
| rs9804922 | 0.09 | 0.911 |
| rs36228499 | 0.44 | 0.983 |
| rs6918698 | 0.46 | 0.112 |
| rs351855 | 0.32 | 0.029 |
| rs3812852 | 0.07 | 0.408 |
| rs974819 | 0.31 | 0.408 |
| rs496339 | 0.10 | 0.951 |
| rs1626340 | 0.21 | 0.008 |
| rs1036095 | 0.25 | 0.964 |
| rs4522809 | 0.45 | 0.536 |
| rs2010963 | 0.34 | 0.099 |
| rs3025039 | 0.13 | 0.598 |
| rs699947 | 0.48 | 0.162 |
| rs5744478 | 0.08 | 0.236 |
| rs1800795 | 0.37 | 0.530 |
| rs1800896 | 0.47 | 0.000 |
| rs3024498 | 0.27 | 0.139 |
| rs1799964 | 0.24 | 0.654 |
| rs1800629 | 0.18 | 0.321 |
| rs361525 | 0.05 | 0.082 |
| rs4986790 | 0.07 | 0.924 |
| rs9527025 | 0.12 | 0.775 |
| rs564481 | 0.39 | 0.257 |
| rs397703 | 0.18 | 0.016 |
| rs577912 | 0.16 | 0.037 |
| rs11574027 | 0.02 | 0.259 |
| rs2238135 | 0.23 | 0.682 |
| rs4516035 | 0.43 | 0.006 |
| rs4918 | 0.31 | 0.610 |
| rs1044291 | 0.33 | 0.296 |
| rs1800787 | 0.21 | 0.606 |
| rs17218711 | 0.15 | 0.783 |

MAF, minor allele frequency, HWE p-value, Hardy Weinberg equilibrium χ^2^ test p-values. P-value <0.05 suggests a disequilibrium.
